# Updated clusters of orthologous genes for Archaea: a complex ancestor of the Archaea and the byways of horizontal gene transfer

**DOI:** 10.1186/1745-6150-7-46

**Published:** 2012-12-14

**Authors:** Yuri I Wolf, Kira S Makarova, Natalya Yutin, Eugene V Koonin

**Affiliations:** 1 National Center for Biotechnology Information, NLM, National Institutes of Health, Bethesda, MD 20894, USA

**Keywords:** Archaea, Orthologs, Horizontal gene transfer

## Abstract

**Background:**

Collections of Clusters of Orthologous Genes (COGs) provide indispensable tools for comparative genomic analysis, evolutionary reconstruction and functional annotation of new genomes. Initially, COGs were made for all complete genomes of cellular life forms that were available at the time. However, with the accumulation of thousands of complete genomes, construction of a comprehensive COG set has become extremely computationally demanding and prone to error propagation, necessitating the switch to taxon-specific COG collections. Previously, we reported the collection of COGs for 41 genomes of Archaea (arCOGs). Here we present a major update of the arCOGs and describe evolutionary reconstructions to reveal general trends in the evolution of Archaea.

**Results:**

The updated version of the arCOG database incorporates 91% of the pangenome of 120 archaea (251,032 protein-coding genes altogether) into 10,335 arCOGs. Using this new set of arCOGs, we performed maximum likelihood reconstruction of the genome content of archaeal ancestral forms and gene gain and loss events in archaeal evolution. This reconstruction shows that the last Common Ancestor of the extant Archaea was an organism of greater complexity than most of the extant archaea, probably with over 2,500 protein-coding genes. The subsequent evolution of almost all archaeal lineages was apparently dominated by gene loss resulting in genome streamlining. Overall, in the evolution of Archaea as well as a representative set of bacteria that was similarly analyzed for comparison, gene losses are estimated to outnumber gene gains at least 4 to 1. Analysis of specific patterns of gene gain in Archaea shows that, although some groups, in particular *Halobacteria*, acquire substantially more genes than others, on the whole, gene exchange between major groups of Archaea appears to be largely random, with no major ‘highways’ of horizontal gene transfer.

**Conclusions:**

The updated collection of arCOGs is expected to become a key resource for comparative genomics, evolutionary reconstruction and functional annotation of new archaeal genomes. Given that, in spite of the major increase in the number of genomes, the conserved core of archaeal genes appears to be stabilizing, the major evolutionary trends revealed here have a chance to stand the test of time.

**Reviewers:**

This article was reviewed by (for complete reviews see the Reviewers’ Reports section): Dr. PLG, Prof. PF, Dr. PL (nominated by Prof. JPG).

## Background

A genome-wide evolutionary classification of genes is essential for the entire enterprise of genomics including both functional annotation and evolutionary reconstruction. The construction of such a classification for a large set of diverse genomes is never an easy task due to the complexity of evolutionary relationships between genes to which gene duplication, gene loss and horizontal gene transfer (HGT) all make major contributions. The interplay of all these evolutionary processes makes accurate delineation of orthologous and paralogous relationships between genes extremely complicated [1-3]. Accurate identification of orthologs and paralogs is central to functional characterization of genomes because orthologs typically occupy the same functional niche in different organisms whereas paralogs undergo functional diversification duplication via the processes of neofunctionalization and subfunctionalization [3-5]. Clear differentiation between orthologs and paralogs is equally important for the reconstruction of evolutionary scenarios [6-9].

In principle, orthologous and paralogous relationships between genes have to be disentangled by means of comprehensive phylogenetic analysis of entire families of homologous genes in the compared genomes [2,10-13]. However, for the case of numerous, diverse genomes, such comprehensive phylogenomic analysis remains both an extremely labor-intensive and an error-prone process. Accordingly, several methods have been developed that aim at the identification of sets of likely orthologs without performing comprehensive phylogenetic analysis; benchmark comparisons indicate that some of these methods perform as well if not, in some cases, better than phylogenomic approaches [1,14-16]. Generally, these non-phylogenomic approaches in orthology inference are based on partitioning graphs of genome-specific best hits for all genes (typically, compared in the form of protein sequences) from the analyzed set of genomes. The key underlying assumption of this approach is that the sequences of orthologous genes are more similar to each other than to the sequences of any other genes from the compared genomes.

The best hit graph approach, supplemented by additional procedures for detecting co-orthologous gene sets and for treating genes encoding multidomain proteins, was first implemented in the Clusters of Orthologous Groups (COGs) of proteins [17]; the acronym COG has been subsequently reinterpreted to simply denote Clusters of Orthologous Genes [3]. The original COG set of 1997 included only 7 complete genomes, all that were available at the time [17]. The latest comprehensive COG collection released in 2003 incorporated ~70% of the protein-coding genes from 69 genomes of prokaryotes and unicellular eukaryotes [18]. The COGs have been extensively used for functional annotation of new genomes (e.g., [19,20], comparative analysis of gene neighborhoods [21-23] and other connections between genes, as implemented in the popular STRING tool [24]; target selection in structural genomics (e.g., [25]); and various genome-wide evolutionary analyses [6,8]. Subsequently, the COGs have been employed as the seed for the EggNOG database that was constructed using improved algorithms for graph-based automatic construction of orthologous gene clusters [26,27].

The methods for the construction of COGs and other, similar clusters of putative orthologous genes cannot guarantee correct identification of the orthologous and paralogous relationships between genes due to the aforementioned complexity of the evolutionary processes. The original COG analysis of small numbers of genomes involved the final step of manual curation that was important for detecting and resolving problems that were not adequately addressed by the automatic procedure. This step ceased to be feasible with the rapid increase in the number of sequenced genomes whereas the computational cost of the analysis has steeply increased. Therefore, along with the development of improved, lower complexity algorithms for identification of orthologous gene clusters [1,15,16], several smaller scale projects have been conducted in which COGs were constructed, annotated and analyzed in detail for compact groups of bacteria such as the *Thermus-Deinococcus* group [28], *Cyanobacteria*[29], and *Lactobacillales*[19]. Along these lines, we have delineated the set of COGs for 41 genomes of archaea [30]; this data set that we denoted arCOGs has become an important tool for archaeal genome analysis [31-34].

Here we present a major update of the arCOGs that includes 120 archaeal genomes and use it for evolutionary reconstructions that seem to provide insights into major trends of archaeal evolution.

## Results and discussion

### Update of archaeal COG database

**Figure 1:**
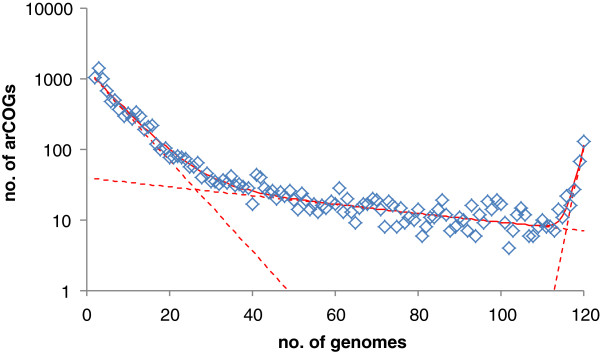
**A commonality plot for Archaeal protein-coding genes.** Diamonds show the number of arCOGs that include the given number of distinct genomes. Dashed red lines, decomposition of the data into three exponents (“cloud”, “shell” and “core” [37,38]); solid red line: the sum of the three components.

The updated arCOG database includes protein sequences from 120 completely sequenced genomes. Altogether, 251,032 protein-coding genes (91% of the total gene complement) were assigned to 10,335 clusters. The coverage of individual genomes by arCOGs ranged from 99% (strains of *Sulfolobus islandicus* and *Methanococcus maripaludis* with abundant close relatives in the set) to 73% (*Nanoarchaeum equitans*, the sole sequenced representative of the phylum Nanoarchaeota). In the current set of archaeal genomes, 129 arCOGs are strictly ubiquitous and 32 more arCOGs are ubiquitous to the exclusion of *N. equitans*; in the original version of the arCOGs, the corresponding numbers were 166 and 50 [30]. With the addition of new genomes, the size of the strictly universal gene set inevitably decreases due to lineage-specific gene losses and possibly also annotation errors [35,36]. In reality, however, the sets of conserved archaeal genes could be stabilizing. Indeed, analysis of the commonality distribution [37,38] for the new arCOG collection gives estimates for the size of the “core” (highly conserved) and the “shell” (moderately conserved) components of the archaeal pangenome that are almost unchanged since 2007 (current estimates of ~220 and ~2,200 vs. ~230 and ~2,200, respectively, for the 2007 arCOG set). By contrast, addition of the new genomes substantially increased (from ~5,200 to ~7,400) the repertoire of rare archaeal genes that belong to the variable “cloud” (Figure 1).

**Figure 2:**
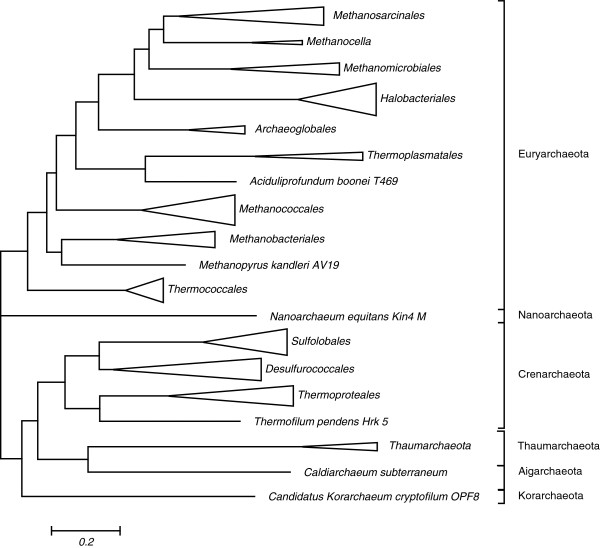
**Phylogeny of universal Archaeal ribosomal proteins.** The approximate Maximum Likelihood tree was reconstructed using FastTree [51,52].

One of the immediate applications of clusters of orthologous genes is phylogenomic reconstruction (i.e. identification of patterns of lineage-specific gain and loss of genes) for the respective group of organisms, in the case of arCOGs, the Archaea. For this reconstruction, a ‘species tree’ is required as a template. We employed the arCOGs of ribosomal proteins to construct a maximum likelihood tree from a concatenated alignment of ribosomal proteins (Figure 2, Additional file 1). Generally, the tree agrees well with the archaeal taxonomy and with the recently published results of phylogenetic analysis including the monophyly of the ‘TACK superphylum’, a large assemblage of archaeal phyla that includes Thaumarchaeota, Aigarchaeota (with the single current representative*, Candidatus* Caldiarchaeum subterraneum), Crenarchaeota and Korarchaeota [39,40].

### Phyletic patterns of arCOGs

**Figure 3:**
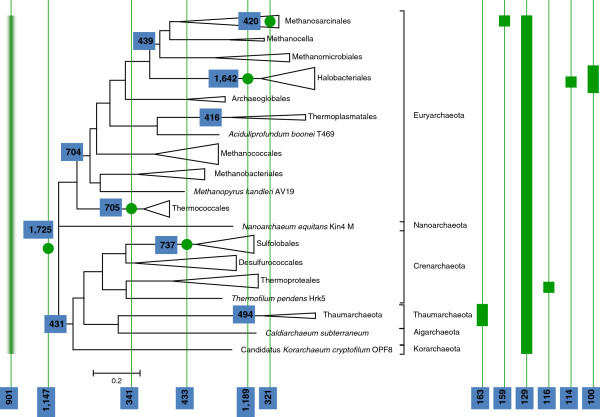
**Phyletic patterns and inferred gene gain patterns in Archaea.** The most frequent phyletic patterns are shown to the right of the tree as blocks of genomes where the gene is present. The inferred number of gene gains is indicated for the tree branches. The most frequent gain patterns are shown as green dots associated with tree branches. The pattern within the *Methanosarcinales* clade refers to the *Methanosarcina* genus. The fuzzy pattern to the left of the tree root refers to the “zero-gain” inference without a confident assignment to any particular clade.

The original 1997 study of the COGs [17] included the first analysis of phyletic patterns, i.e. patterns of presence-absence of genes from a given COG in the genomes of the analyzed organisms. Subsequently, phyletic patterns proved useful in describing the evolutionary history of lineages and functional relationships between genes [41-46]. The current set of 10,335 arCOGs includes 6,736 phyletic patterns of which 5,998 (89%) are unique. Similar to our 2007 observations, we found that the most common patterns represent genes that are conserved in well-defined archaeal clades, such as all 120 Archaea, 4 Thaumarchaeota or 3 species of *Methanosarcina* genus (Table 1 and Figure 3).

**Table 1:** Phyletic patterns in arCOGs

**Pattern frequency**	**Pattern description**	**No. of Species**
163	all Thaumarchaeota	4
159	all Methanosarcina	3
129	all Archaea	120
116	all Pyrobaculum + Thermoproteus	7
114	all Halobacterium	2
100	all Halobacteriales	16

Analysis of the current arCOG set reveals the fundamental limitation of phyletic patterns as the basis for phylogenomic analysis. The early, small genomic data sets were readily amenable to direct pattern counting (e.g. in the original 1997 COGs that included 7 species, about 1/3 of the possible patterns with 3 or more species were found in the actual data), the current set of 120 archaeal species allows for ~10^36^ possible patterns of which only a tiny fraction (~1/10^32^) are actually observed. An overwhelming majority of the observed patterns are unique and even the most frequent patterns represent at most 1.6% of the arCOGs. Even under unrealistically restrictive models of gene content evolution that prohibit horizontal gene transfer, random loss of non-essential genes alone results in an exponential decrease in the number of non-unique phyletic patterns with the number of genomes in the data set.

The rapidly increasing proportion of unique phyletic patterns calls for a more coarse-grained comparison whereby non-identical but similar patterns are treated as members of the same group. However, standard clustering and ordination techniques perform poorly on phyletic pattern data because of the difficulty of objectively assessing the similarity between the observed phyletic patterns of (ar)COGs. A proper similarity measure must take into account relationships that go beyond simple distances between 120-dimensional binary vectors primarily because different dimensions are not independent or correlated in a simple manner, but are connected by a complex network of phylogenetic and environmental relationships between the corresponding organisms. As a step toward a more biologically meaningful analysis of phyletic patterns, we compared evolutionary scenarios that are implied by these patterns.

### Gains, losses and ancestral states in arCOGs

**Figure 4:**
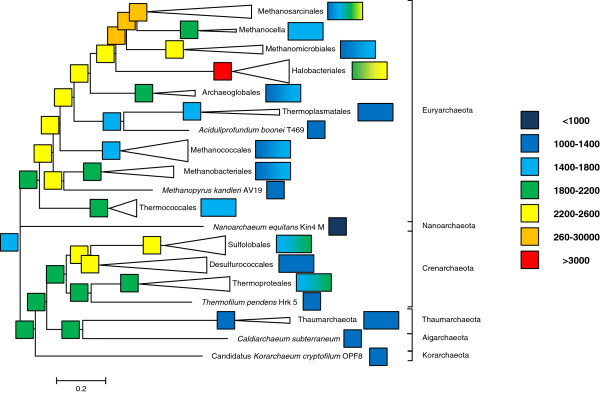
**Inferred ancestral genomes in Archaea.** The square boxes at the bases of clades indicate the number of families in the inferred ancestral genomes; the rectangles at the tips of clades indicate the number of families in the extant genomes within the clade. Square boxes at the tips indicate single genomes; rectangular boxes indicate multiple genomes.

We reconstructed the posterior probabilities of gene presence in ancestral nodes and the probabilities of the associated gene gain and loss events using the Count method of Csűrös and Miklós [9]. The reconstruction was based on binary patterns (i.e. ignoring paralogs), and the topology of the ribosomal protein tree (Figure 2) was used as the guide. The results of the present reconstruction (Figure 4) that was based on 10,335 arCOGs represented in 120 species of archaea generally agree with the earlier observations made with the original arCOG collection using maximum parsimony [30] as well as the outcome of the ML reconstruction reported by Csűrös and Miklós [9]. The genome of the last archaeal common ancestor (LACA) is inferred to have contained genes from at least ~1725 arCOGs of which 970 could be identified with high confidence (posterior probability >0.9). Taking into account the characteristic level of paralogy and the number of genes in the transient “cloud” (Figure 1), the genome size of LACA can be estimated at around 2600 genes, which puts this ancestor form on the high end of genomic complexity by the standards of the extant archaeal genomes (Additional file 2). The history of most archaeal lineages appears to have involved gradual, moderate genome growth or genomic stasis in the deep branches followed by extensive gene loss (genome streamlining) during the diversification of recent family-level groups (Figure 4).

Gene gains that are associated with the ancestor of a clade conceivably reflect the innovations that led to the diversification and define the biological characteristics that differentiate the given clade from other groups. Among the Archaea, the maximum number of gene gains was detected at LACA (Table 2 and Figure 3); these gains necessarily include both genes inherited from the last universal common ancestor of cellular life and genes acquired during the stem phases of archaeal evolution. Among the internal branches of the archaeal tree, the branch leading to the common ancestor of *Halobacteriales* is associated with >1600 gene gains, bringing the inferred size of the ancestoral halobacterial genome to >3000 genes. Other branches characterized by high rates of gene gain include Sulfolobales, Thermococcales, Methanomicrobia and others. Of special interest is the gain of 431 genes assigned to the common ancestor of the proposed TACK-superphylum. Although the inference of gene gain depends on tree topology and therefore cannot be construed as direct evidence of the monophyly of any group, such a high number of gains indicates a strong signal of shared gene content among the archaeal phyla that constitute the TACK superphylum.

**Table 2:** Inferred gene gains in Archaea

**No. of gains**	**Clade**
1,725	LACA
1,642	Halobacteriales
737	Sulfolobales
705	Thermococcales
704	deep Euryarchaeota (Eury- without Thermococcales)
494	Thaumarchaeota
439	Methanomicrobia
431	TACK superphylum
420	Methanosarcina
416	Thermoplasmatales

### Comparison of the rates of gene gain and loss in Archaea and Bacteria

For comparison with Archaea, we analyzed the phyletic patterns of 50 bacterial species in the 2003 COG data set [18]. The evolutionary scenarios were reconstructed using the Count software with the same parameters as used for Archaea and the ribosomal protein phylogeny [39] as the guide tree topology (Table 3). Despite the difference in sampling density and breadth (the COG set of 2003 covers a relatively small fraction of the bacterial diversity), the estimated numbers of gene losses per COG and the gain-loss ratio are very similar for the two prokaryotic domains of life. Strikingly, Archaea and Bacteria display essentially the same, four-fold excess of losses over gains. For Bacteria, the reconstruction yielded 20-25% more secondary gene gains (in addition to one default gain in the respective last common ancestor) per COG (0.87 in bacteria vs. 0.71 in archaea) and a higher fraction of COGs with multiple (estimated total number >1.5) gene gains (47% in bacteria vs. 39% in archaea). Thus, although the overall modes of genome-scale evolution are similar in both prokaryotic domains, intra-domain gene exchange seems to have played a greater role in the evolution of bacteria. This finding does not appear surprising given the typical greater complexity of bacterial compared to archaeal communities.

**Table 3:** Comparative analysis of gene gains and losses in Archaea and Bacteria

	**arCOGs**	**bac. COGs (2003)**
families	10,335	4,149
species	120	50
gains	17,680	7,769
gains/family	1.71	1.87
acquisitions/family	0.71	0.87
losses	74,690	32,355
losses/family	7.23	7.80
loss/gain ratio	4.22	4.16
single-gain (<1.5)	61%	53%

### Patterns of gene gain in Archaea

Phyletic patterns form in the course of evolution by gene gain (largely via HGT), vertical transmission through speciation, and gene loss. Of these processes, gene gain seems to be of greatest interest. As shown above, gene loss is much more frequent, appears to be largely random in terms of which clades are affected and generally appears to occur at approximately constant rate over long spans of evolution (a form of molecular clock) [6]. Thus, we focus here on the patterns of gene gain, ignoring possible subsequent gene loss.

Unlike parsimony-based methods that reconstruct binary evolutionary scenarios, Count produces profiles of posterior probabilities of events. To convert these into patterns, we use the simple definition of a “likely event”: if a gene gain probability is >0.5 for a particular branch, we mark it as a likely gain. Thus, instead of 120-dimensional (given 120 analyzed genomes) binary presence-absence patterns, we obtain 238-dimensional (the tree has a total of 238 branches, including the branch leading to the root) binary gain patterns.

Remarkably, despite the near doubling of the dimensionality, the likely gain patterns provide considerably more coarse-grained material for comparisons. The 10,335 arCOGs form 1,878 gain patterns, of which 77% are unique (compared to 6,736 presence-absence patterns with 89% of them being unique). The most common (apart from the obvious overall winner, the single gain in LACA) gain pattern (Figure 3 and Table 4), a single gain in the Halobacteriales ancestor, is inferred for 1,189 arCOGs. With one exception, all frequent (>300) gain patterns involve a single gain. The only exception is the “zero-gain” pattern that was assigned to 901 arCOGs. These genes are scattered among archaeal clades in such a way that COUNT could not confidently assign a gain to any particular branch (e.g. arCOG11374 is found in *Methanobacterium sp.* AL-21 and in *Methanosphaera stadtmanae* DSM 3091 that are neither sister nor very distant species; informally, it appears likely that this gene has been transferred from bacteria to some *Methanobacteriaceae* ancestor).

**Table 4:** Inferred gene gain patterns in Archaea

**Pattern frequency**	**Pattern description**	**No. of Species**
1,189	Halobacteriales	16
1,147	LACA	120
901	scattered (no confident gains)	N/A
433	Sulfolobales	13
341	Thermococcales	12
321	Methanosarcina	3

**Figure 5:**
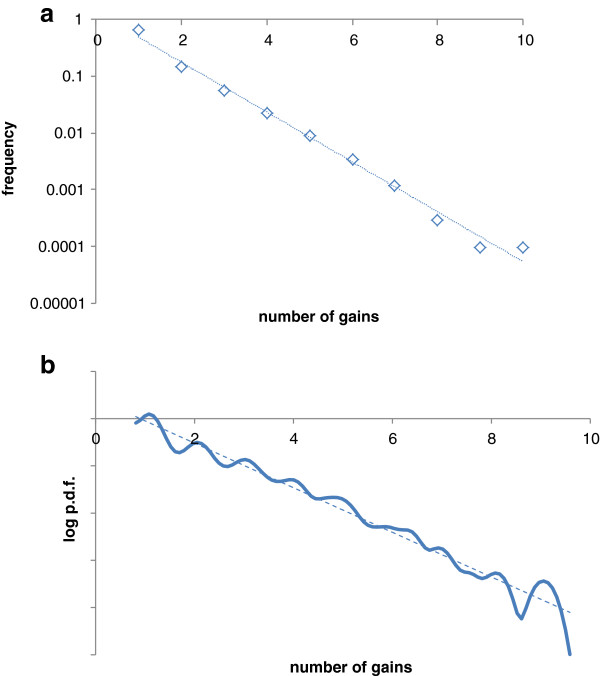
**Distribution of the gain patterns by the number of gains. a.** Number of confidently predicted gains in a pattern (*p* > 0.5). **b.** The sum of posterior gain probabilities in a pattern (kernel-smoothed probability density). Dotted line, the best-fitting exponent.

The frequency distribution of the number of gains per arCOG, plotted either in the discrete form for likely gains (Figure 5a) or in the continuous form for the sums of posterior probabilities (Figure 5b), indicates that the single-gain patterns represent 61-67% of the data and the number of arCOGs with multiple acquisitions declines exponentially. The excellent fit of the number of gene gains to the exponential decay function implies that horizontal transfer of genes of each arCOG occurs in a random fashion.

### Multiple gene gains in archaea

The inferred multiple gene gains in the same arCOG could provide insights into the history of intra-domain gene exchange. Altogether there are 2,495 arCOGs with ≥2 likely (posterior probability >0.5) gains (excluding those with a gene gain in LACA). These families are implicated in a total of 6,190 gene acquisitions.

Over 20% of the multiple gains involve the ancestral branch of the *Halobacteriales* (Table 5) which is the absolute leader among Archaea with respect to involvement in gene exchange as either the donor or the acceptor. Other prominent gene exchange participants are *Thermococcales, Thermoplasmatales* and *Sulfolobales*. Generally, the number of multi-gain events on archaeal tree branches strongly correlates with the overall number of gains (*r*_*S*_ = 0.83, *p* < 0.0001 for the entire tree; *r*_*S*_ = 0.93, *p* < 0.0001 for the internal branches; 36% of the internal branch gains involve multiple branches) (Additional file 3). This observation, again, is compatible with a largely random gene exchange. Nevertheless, some branches appear to significantly deviate from the expected behavior: for example, 94 of the 109 arCOGs likely acquired by the common ancestor of Thaumarchaeota and Aigarchaeota were also acquired elsewhere, which is over than twice more than expected; conversely, on the *Pyrobaculum*-*Thermoproteus* clade, the number of multiple-event gains was almost twice less than expected (54 out of 265).

**Table 5:** Inferred gene gains in Archaea

**No. of gains**	**Clade**
565	Halobacteriales
296	Thermococcales
247	Thermoplasmatales
245	Sulfolobales
204	Thaumarchaeota
199	deep Euryarchaeota
166	Archaeoglobales
123	TACK superphilum
104	Methanobrevibacter ruminantium
98	Methanosarcina

### Routes of gene exchange in Archaea

**Figure 6:**
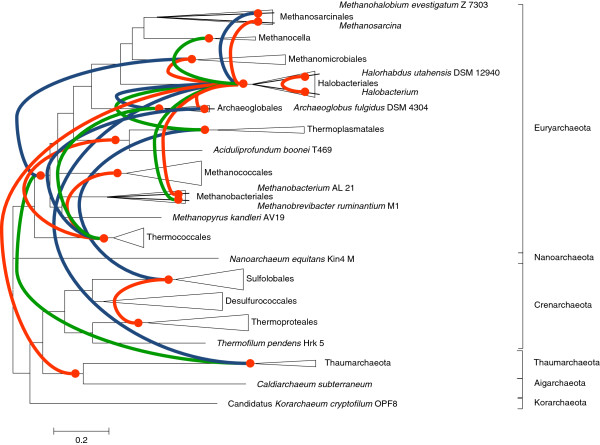
**The byways of horizontal gene transfer among Archaea.** Lines connect the clades that form the most frequent phyletic patterns with two inferred gains (different colors are used for visual differentiation only). One of the two clades is the likely origin of the respective arCOG and the other is the likely acceptor of the HGT.

To explore the preferred routes of intra-archaeal gene exchange, we focus on the 1,267 arCOGs with 2 predicted gains not involving LACA. This pattern likely indicates a single gene exchange within archaea (or alternatively, two independent acquisitions by different archaeal clades from outside of the domain; however, this is a distinctly less parsimonious solution). The most frequently occurring 2-gain pattern (Table 6, Figure 6, Additional file 4) is the *Thermoplasmatales-Sulfolobales* pair, which was found in 16 arCOGs; an unexpectedly large number of shared genes between *Sulfolobus* and *Thermoplasma*, suggestive of preferential HGT, has been reported previously [47]. Not surprisingly, many pairs of clades frequently exchanging genes involve *Halobacteriales*, the archaeal group that seems to be generally most prone to HGT (see above).

**Table 6:** Inferred gene gain patterns involving 2 Archaeal clades

**Pattern frequency**	**Pattern description**
16	Thermoplasmatales – Sulfolobales
14	deep Euryarchaeota – Thaumarchaeota
13	Halobacteriales – Thaumarchaeota
13	deep Euryarchaeota – Methanomicrobiales
12	Thermoplasmata – Thermococcales
12	Halobacteriales – Methanocella
12	Halobacteriales – Thermoplasmatales
12	Halobacteriales – Methanohalobium evestigatum
11	Halobacteriales – Archaeoglobus fulgidus
11	Halobacteriales – Archaeoglobales
10	Halobacteriales – Thaum- + Aigarchaeota

If the paths of gene exchange are random, the number of exchanges between a pair of clades is expected to be proportional to the product of the numbers of gains on these clades. We found that these variables were indeed correlated, relatively weakly but significantly (*r*_*S*_ = 0.39, *p* < 0.0001 for 196 pairs occurring more than once). However, the most frequently observed exchanges between clade pairs are an order of magnitude more frequent than expected by chance, an overwhelmingly unlikely fluctuation for the majority of these patterns (Additional file 4). Taken together, these findings indicate that, although HGT between the lineages was not completely random, no major “highways” [48] of intra-domain gene exchange existed in the history of archaea. At best, there seem to exist weakly preferred “byways” of HGT.

## Conclusions

The updated version of the arCOG collection incorporates 91% of the pangenome of 120 archaea into 10,335 arCOGs. This new set of arCOGs is expected to become a key resource for comparative genomics, evolutionary reconstruction and functional annotation of archaeal genomes that undoubtedly will be appearing at an increasing pace. Notably, between this new arCOG collection and the original, 2007 version, the conserved gene core of Archaea has not substantially shrunk, suggesting that the present composition of this core could be close to definitive. We describe here some results of the ongoing work on reconstruction of the genome content of archaeal ancestral forms and gene gain and loss events. This reconstruction clearly indicates that the last common ancestor of the extant Archaea was a complex organism, most likely with over 2,500 genes, and that the principal trend in subsequent evolution of almost all archaeal lineages was gene loss leading to genome streamlining. Overall, in the evolution of Archaea as well as a representative set of bacteria that we analyzed for comparison, gene losses are estimated to outnumber gene gains at least four to one. We further investigated the specific patterns of gene gain in Archaea and found that, although some archaeal groups, in particular Halobacteria, are more prone to gene acquisition than others, on the whole, gene exchange within Archaea appears to be largely random, so that there are no major ‘highways’ of gene transfer.

The arCOG data is available at <ftp://ftp.ncbi.nih.gov/pub/wolf/COGs/arCOG/>.

## Methods

### Construction of archaeal COGs

Protein sets for 120 completely sequenced genomes of Archaea were downloaded from the NCBI FTP site. New members of the previously established arCOGs were identified using the PSI-BLAST search with core arCOG alignments as the PSSMs. New arCOGs were constructed largely as previously described [30]. Briefly, the procedure involved the following steps:

• Initial clusters based on triangles of symmetrical best hits were constructed using a modified COG algorithm [1] on the results of all-against-all BLAST [49] search.

• Multiple alignments of the initial cluster members constructed using the MUSCLE program [50] were used as PSSMs for a PSI-BLAST search [49] against the database of Archaea proteins; significantly similar proteins (domains) were added to the corresponding original clusters.

• The clusters with approximately complementary phyletic patterns and high inter-cluster sequence similarity were merged.

### Phylogenetic analysis

The maximum likelihood phylogenetic tree of the concatenated alignment of ribosomal proteins universal for archaea was constructed using the FastTree program [51,52] as previously described [39].

### Reconstruction of gene gain and loss events during the evolution of Archaea

Reconstruction of gene gain and loss during the evolution of Archaea was performed using the program COUNT [53]. Maximum likelihood inference for gene presence, gain and loss at all branches of the phylogenetic tree was obtained for 2-category evolutionary model.

## Competing interests

The authors declare that they have no competing interests.

## Authors’ contributions

KSM and YIW collected the data; KSM, NY, EVK and YIW analyzed the data; YIW and EVK wrote the manuscript that was read and approved by all authors.

## Reviewers’ reports

Reviewer 1: Dr. Purificacion Lopez-Garcia, Centre National de la Recherche Scientifique, France

This manuscript presents an update of the archaeal cluster of orthologous genes (arCOGs) based on 120 archaeal genome sequences which, although still limited face to the wide archaeal diversity, is a large improvement compared to the initial set of genomes used to define the first arCOGs. The arCOGs become therefore much more comprehensive and, on this sole basis, the work is already important and useful. In addition, the authors take advantage of this analysis to make some inferences about archaeal genome evolution. The authors estimate in this way the number of genes at ancestral nodes (including the last common archaeal ancestor, LACA) and the estimated number of gene gains at different nodes on a guide phylogenetic tree based on highly conserved ribosomal proteins. The number of genes shared by distant archaeal groups is used to evaluate the level of intra-domain gene transfer and compared with that observed in a (smaller) set of bacterial genomes. Overall, I find the article interesting and useful.

I have a few comments that the authors might want to address:

- Wolf et al reconstruct an archaeal phylogeny based on the arCOGs of ribosomal proteins as guide for subsequent inferences of genome evolution. According to the authors the tree agrees with known taxonomic schemes and points to the monophyly of the “TACK” supergroup (Thaumarchaeota –Aigarchaeota – Crenarchaeota - Korarchaeota). This is difficult to say from an unrooted tree, because the root might lie somewhere else in the tree, e.g. between the Korarchaeota and the remaining “TAC” or elsewhere, in the presence of an appropriate outgroup. At any rate, the TACK monophyly appears to be supported by a large number of shared genes (431). At this point, it may be timely to consider a more systematics-oriented comparison in terms of the scale of gene loss and gain and genome evolution between the Euryarchaeota and the “TACK” super-phylum. Are we in front of comparable evolutionary scales in terms of phylogenomic breadth? If that is the case, could the TACK super-phylum be assimilated with Crenarchaeota sensu lato? In other words, could we define a less arbitrary barrier for the phylum level within the archaea based on phylogenomic information? A comment along those lines would be welcome.

Authors’ response: *We cannot and should not address issues of formal taxonomy in this article. What really matters is the cladistic pattern; which clades are given the status of “phylum” and which are subsumed within other phyla matters only operationally.*

- The authors detect a higher intra-domain transfer in bacteria than in archaea, and do not find it “surprising given the typical greater complexity of bacterial compared to archaeal communities”. However, it is not self-evident that greater community complexity necessarily correlates with more horizontal gene transfer. Most likely, there is an important phylogenetic effect, with some lineages being more prone than others even within bacteria. The observation of a higher intra-domain transfer in bacteria could be biased by the taxonomic sampling and also the more limited sampling size for bacteria. Can these possibilities be discarded?

Authors’ response: *The possibility that the results of any comparative genomic analysis are affected by taxonomic sampling bias effectively can never be discarded given that the existing samples represent a tiny slice of the entire existing biodiversity. Nevertheless, some of the sampling bias effects can be mitigated by using the tree-based probabilistic inference of ancestral states and evolutionary events (e.g. the potential effect of the sampling bias introduced by inclusion of 7 strains of* Sulfolobus islandicus *in our data set is reduced by the fact that their gene complements are not counted independently but are largely shared by the common branch of* S. islandicus *and* S. solfataricus*). Regardless, we trust that this comment and response will remind the reader of inevitable incompleteness of comparative genomic analyses.*

- The number of inferred ancestral genes for LACA, but also for the last common bacterial ancestor is relatively high and the number of gene losses outnumbers gene gains by at least a factor of 4. Given the importance of horizontal gene transfer in prokaryotic evolution and the fact that many genes evolve by duplication and neofunctionalization, the idea of a gene-rich ancestral genome evolving by important gene loss seems a bit odd. Is this real or does this result from our incapacity to identify gene transfer events and/or failure to identify some type of gene coalescent processes in the past? What are the potential biases of this inferred value?

Authors’ response: *The idea of a deep ancestor that was more complex than an average modern member of the descendant clade probably is “*a bit odd*” (or even more than a bit) to many biologists. We submit, however, that this oddity is primarily a vestige of the traditional belief in the increase of complexity with evolutionary time, a notion that might be intuitive but is not actually borne by any substantial evidence*[54]*. Given the importance of genome streamlining, especially in the evolution of prokaryotes, there is no reason to a priori dismiss the possibility that gene loss on average outweighs gene gain caused by HGT as well as duplication followed by neo/subfunctionalization (the latter process being relatively less important in prokaryotes). The inference of the complex ancestor is “real” in the sense that this is the maximum likelihood estimate based on the best possible estimation of the parameters of the evolutionary process (gene gain and loss rates) from the available data on phyletic patterns. Thus, HGT is taken into account implicitly here, under the gene gain rate. What is meant by “*some type of gene coalescent processes in the past*” is not quite clear to us. Admittedly, the current inference methodology is somewhat crude, being based on phyletic patterns and not taking into account the phylogenies of individual genes. Such a comprehensive analysis is beyond the capabilities of the currently accessible maximum likelihood models. However, given the apparent (approximate) randomness of the HGT processes, it appears highly unlikely that if and when such analysis becomes feasible, it changes the conclusions qualitatively. With regard to the possible biases, this again goes back to the taxonomic sampling and its potential incompleteness. If tomorrow a deep branch of Archaea with very small genomes is discovered, this will result in a downward reassessment of the complexity of LACA.*

*The inference of complex deep ancestors is neither entirely new nor limited to the evolution of Archaea. Many evolutionary reconstructions and theoretical models of genome evolution point to extensive gene loss as one of the key evolutionary processes*[55-57]*. Perhaps particularly impressive are reconstructions of the ancestral gene structure in eukaryotes that suggest highly complex, intron-rich ancestral genomes*[58,59]*. Generally, it appears to be a distinct possibility that genome degradation and streamlining is one of the quantitatively dominant modalities of evolution.*

Minor spelling errors:

Page 7, bottom, Thaumarchaeota (not Taumarchaeota), Candidatus (only Candidatus in italics) Caldiarchaeum subterraneum (not subterreneum)

Authors’ response: *Errors fixed.*

Reviewer’s response: Dear Editorial Team,

I appreciate the response of Wolf et al. and find their manuscript suitable for publication. However I have an additional comment as follows: It seems to me that the authors are avoiding answering my previous question "Are we in front of comparable evolutionary scales [between Euryarchaeota and the TACK superphylum] in terms of phylogenomic breadth?" This is not a taxonomy question. It is a comparative genomics question whose answer might have implications for Systematics (it would correspond then to taxonomists to take this information into account for their work), but not exclusively. It would also be important to determine patterns and eventually unravel processes of archaeal evolution within the two major clades put forward by the authors. Actually, Wolf and colleagues insist in the number of shared arCOGs as a signature for the TACK superphylum, but then refrain themselves from going further and explicitly make a direct comparison with the situation in the Euryarchaeota.

The authors may choose not to comment on this and stay on the simple description of arCOGs and their distribution (which is already very useful). However, this is a pity in when they have the data at hand. It is also at odds with the more general ambition of the manuscript to extract evolutionary information about archaeal genome evolution (which they do when focusing e.g. on horizontal gene transfer and gene gain/loss).

Authors’ response to reviewer’s response: *We appreciate the importance of the reviewer’s question but continue to believe that this is outside of the immediate scope of our work.*

Reviewer 2: Prof. Patrick Forterre, University Paris XI, France

The archaeal domain is presently the only one for which a robust evolutionary history based on phylogenies of large concatenations of protein markers is available [60]. This is probably because the number of currently sequenced genomes is not yet very large, or because the different archaeal phyla and orders diverged less rapidly than bacterial and eukaryal ones, reducing the “radiation” effect that makes difficult the resolution of the most basal nodes in the global bacterial and eukaryal phylogenies. Therefore, I was really interested to read this paper describing comparative genomic results obtained from an updated version of the arCOGs database. The new database contains 120 archaeal genomes, a significant improvement compared to the 41 genomes used in the first version (2007). The results obtained are in line with previous ones [9,30], suggesting a rather complex Last Archaeal Common Ancestor (LACA) and an important streamlining component in archaeal evolution, since gene losses outnumber gene gains by at least four to one.

An essential starting point of all analyses that seek to estimate ancestral gene contents, and subsequent gene losses and gene gains is the availability of a solid reference phylogeny. Therefore, I have two important concerns on this analysis:

1) Concatenation of ribosomal proteins has been widely used over the past ten years to reconstruct a robust reference phylogeny of the Archaea [60]. Therefore, it seems of no use to recalculate it, all the more if this is done by using a fast and approximate tree-building algorithm. The authors should rather use a sketched phylogeny that makes a consensus of previous analyses. The tree presented here is probably not the best one, as suggested by the branching of Nanoarchaeota between Euryarchaeota and other Archaea. *Nanoarchaeum equitans* is more likely a fast evolving Euryarchaeote probably related to Thermococcales [60,61]. In addition to its position in the updated ribosomal protein trees of Brochier et al. [60], the grouping of Nanoarchaeota with Thermococcales is supported by several other phylogenies, as well as a clear-cut synapomorphy corresponding to the transfer of a bacterial tRNA modification enzyme to the Nanoarchaea/Thermococcales clade [61,62].

Authors’ response: *We fail to see good reasons to use a “sketched phylogeny” from previous studies. Our phylogenetic reconstruction utilizes the largest and most thoroughly curated data set available to date and employs a powerful and robust phylogenetic method*[51,52]*that, in our experience, produces better results than most allegedly “precise” ML implementations. The results described here fully agree with our previously published reconstructions*[39]*. We believe that, although many archaeal branches are well established so that the results of the reconstruction should not dramatically depend on the tree topology, the strategy that we used is the best current choice to optimize the reconstruction.*

2) Even more problematic, the authors have artificially puts the root at the junction point between Nanoarchaeota, Euryarchaeota and the recently proposed “TACK superphylum”, i.e. a group encompassing Thaumarchaeota, Aigarchaeota, Crenarchaeaota, and Korarchaeota [40]. However, because the rooting of the archaeal tree has not been firmly established, the very existence of the “TACK superphylum”, -since it implies to root the archaeal tree in the euryarchaeal branch- remains to be confirmed [63]. Therefore, the choice of rooting the archaeal tree between the “TACK superphylum” and Euryarchaeota appears arbitrary, and the authors should definitely test alternative rootings, i.e. in all branches leading to the major archaeal phyla (T-C-K-E).

Authors’ response: *Given the high computational demands on the ancestral reconstruction method (more than a week of wall-clock time), we find it impractical to test multiple root positions. See below the argument for our choice of the root position for the purpose of reconstruction.*

I would be especially interested by comparative analyses based on rooting the archaeal tree in the branch leading to Thaumarchaeota (including Aigarchaeota, see below). This rooting was obtained for the ribosomal protein tree using a eukaryotic outgroup [64]). Such rooting makes sense, considering that Thaumarchaeota exhibits several important eukaryotic features that are not present in other Archaea (Topo IB, a monomeric RNA polymerase A subunit, the presence of both RPA and “cren” SSB proteins) [64,65]. The use of eukaryotes as an outgroup to root the archaeal tree is reasonable since eukaryotes most likely emerged either from a deeply branching archaea or from a proto-eukaryote, but not within lineages leading to modern Archaea. Indeed, although the situation is not yet settled, it is difficult to imagine the emergence of eukaryotes from a reduced organism, such as a modern Archaea or even LACA. I know that some phylogenetic analyses suggest that Archaea emerged from within Archaea. However, I don’t trust these analyses. The use of universal proteins to root the archaeal tree is problematic because archaeal and eukaryotic proteins are quite divergent from their bacterial homologues. As a consequence, archaeal rootings obtained with universal trees are often contradictory and cannot be taken for granted [66].

Authors’ response: *The reviewer briefly but impressively describes the multiple controversies around the topology of the archaeal tree and the position of eukaryotes. Being well aware of these problems, we deliberately chose to avoid adhering to any particular hypothesis and instead to root the archaeal tree in the least controversial position. Both Euryarchaeota and the TACK members have a large arCOG repertoire specific to the respective group and hence representing likely derived shared characters (32 arCOGs are present in >90% of Euryarchaeota to the exclusion of TACK and 73 arCOGs are present in >90% of Crenarchaeota and all Thaum-, Aig-and Korarchaeota to the exclusion of Euryarchaeota). In our reconstruction, Nanoarchaeon is derived directly from LACA, minimizing the effect of its highly reduced gene repertoire on the reconstruction of the other clades.*

In any case, since their phylogeny is arbitrarily rooted, the authors cannot claim that their analysis confirms the existence of a monophyletic “TACK” superphylum!!

Authors’ response: *Indeed, we point out in this article that “*the inference of gene gain depends on tree topology and therefore cannot be construed as direct evidence of the monophyly of any group”*. Nevertheless, we indicate that the large number of shared gene gains makes the monophyly of the TACK a plausible hypothesis (in a weak Popperian sense, the results of our analysis fail to provide any evidence against the TACK monophyly).*

The name TACK itself raises problem because, in my opinion, Aigarchaeota (A) should be better considered as an order of the phylum Thaumarchaeota. Caldarchaeum subterraneum, the only member of this new order indeed forms a robust monophyletic group and share similar genomic signatures with other Thaumarchaeota [67]. If the status of phylum is delivered to “Aigarchaeota”, Crenarchaeota and Euryarchaeota should be divided into several new phyla, since Thaumarchaeota and “Aigarchaeota” are more closely related in term of distance than several internal groups of Crenarchaeota and Euryarchaeota in ribosomal protein trees [60].

Authors’ response: *This is an issue of formal taxonomy. We have neither intent nor authority in this paper to offer any judgment on the appropriate taxonomic level of any of the (putative) major archaeal clades discussed here. We refer to “Aigarchaeota” (following the literature*[40,68]*) for convenience of discussion of the hypothetical TACK superphylum.*

Finally, I think a bit confusing to talk of gene gain in the case of LACA (the obvious overall winner). Of course, starting from the origin of life, gene gain obviously occurred before any streamlining. However, talking about gene gain for LACA seems implying that LACA was positioned exactly at the transition point between gene gains and gene losses in the evolution of the archaeal (and pre-archaeal) lineages. This would not be correct. In fact, LACA was no more a transition point in archaeal evolution than the African eve in Homo sapiens evolution. The streamlining tendency observed by Wolf and colleagues (and others) in archaeal evolution probably taken place not only between LACA and modern Archaea, but between the last common ancestor of Archaea and Eukarya (LCAAE) and modern Archaea. For me, this fits well with the idea that reductive evolution was instrumental in shaping the archaeal (and bacterial genomes) by streamlining starting from a more complex LCAAE, possibly via thermoreduction [69,70].

Authors’ response: *The gene repertoires of both LUCA and LAECA/LCAAE (if such an organism existed outside of the diversity of modern Archaea) are outside the scope of the present work. We do not assign “*transition point*” status to any of the common ancestors of the observed clades; the ancestors simply are operationally defined by the relationships between these clades themselves. Formally, the genes that “appear” in LACA under our reconstruction have to come from somewhere; thus, they are considered gains in LACA in a purely formal sense, regardless of their history outside of the modern Archaea.*

Finally, the work of Wolf and colleagues reveals that HGTs appears to have occurred largely randomly during the evolution of Archaea, with few exceptions. This again shows that HGTs are not a major problem for tree reconstruction. This is refreshing in the framework of the debate between tree-thinkers and web-thinkers [71]. However, the existence of a few exceptions” reminds us that one should be careful (taking these exceptions into account) in performing global phylogenetic analyses based on whole genome trees.

Reviewer’s response: I understand that it would be impractical for this particular work to test multiple root positions and I suspect that the result should not be very different. I hope that for the next update, it will be possible to use a well supported rooting, beyond the “TACK superphylum”.

I also can understand that the authors prefer to use their phylogenetic reconstruction. However, in that case, I would like to remind that recovering the best tree is not only the problem of the phylogenetic method used, but of the critical incorporation in a consensus phylogeny of different information (especially removing genes affected by HGT and fast evolving lineages at once to test different positions). For instance, to recover the “most likely” correct position of Nanoarchaea, it was first necessary to analyze all individual ribosomal protein phylogenies to realize that the correct result was probably the grouping of nanoarchaeota + Thermococcales [61]. This grouping was then support by additional phylogenies (reverse gyrase, Topo VI, elongation factors) and a synapomorphy based on a tRNA modification protein. It’s the reason why I think that a consensus phylogeny based on the work of several groups should be preferred for the kind of work presented here. In any case, the update arcCOG database will be a powerful addition to genomic tools available to study archaeal evolution and I am fine with the revised version.

Authors’ response to reviewer’s response: *We will definitely revisit the question of the archaeal core phylogeny when working on the next arCOGs update. We would like to add a word of caution regarding consensus phylogenies: there is a body of evidence indicating that the “supermatrix” approach (concatenated alignments) provides more robust phylogenetic reconstructions compared to the “supertree” (consensus) methods*[72-75]*.*

Reviewer 3: Dr. Pascal Lapierre, University of Connecticut, USA (nominated by Prof. J Peter Gogarten, University of Connecticut, USA)

Review for the manuscript titled “The updated clusters of orthologous genes for Archaea: a complex ancestor of the archaea and the byways of horizontal gene transfers”

This manuscript by Wolf et al. is presenting the updated version of the clusters of orthologous genes for Archaea (arCOGs), as well as a detailed analysis of the genomic evolutionary history of the archaeal domain. This new version of arCOGs now includes data from 120 archaeal genomes (up from 41), segregated into 10,335 clusters of orthologous genes. Based on the genomic distributions of these gene families, Wolf et al. were able to determine that the last common archaeal ancestor was more complex and that genome streamlining lead to the smaller genomes found in most of the modern archaea. They also found that gene gain through horizontal gene transfers across archaeal species did not shown any preferred highway of gene sharing. Cluster of orthologous gene databases have been proven to be a valuable tool for genomic analysis. This updated version will help increase the accuracy and reliability of genome annotation and functional and comparative genomics. There are however, a few questions and concerns about the analysis and conclusions in this paper.

1) Regarding the arCOGs core and shell compositions, the authors are saying that the archaeal “core” genome went from 230 arCOGs in 2007 to 220 arCOGS, while the number of arCOGs present in the “shell” stayed unchanged to about 2000 arCOGs. What happened to the 10 extra core arCOGs that were present in 2007? Shouldn’t they have move to the “shell” category if they were no longer highly conserved amongst the archaeal genomes?

Authors’ response: *The numbers are approximate (see below on the nature of the estimates) and are given to the 2 significant digits. Although the 10 arCOGs that disappeared from the core, indeed, most likely moved to the shell category, the size of the latter partition remains ~2200 because reporting it as 2210 would be excessively precise and beyond the power of the present analysis.*

2) On the estimation of LACA genome size, I found conclusions based on phyletic patterns are difficult to accept, mainly because ancestral genomes reconstruction always tends to be larger than the extant genomes. If HGTs are rampant and probably were so in the past, the phyletic patterns that we see today for most of the proteins impacted by frequent transfers will fool any attempt to accurately reconstruct ancestral genome history. Can the authors comment on this?

Authors’ response: *It is not the case that “ancestral genomes reconstruction always tends to be larger than the extant genomes”. This is observed only when gene loss is arbitrarily assumed to be more common than gene gain (in particular, via HGT)*[8]. *Quite the contrary, simple reconstruction methods such as maximum parsimony tend to yield simple ancestors. That said, theoretically, the situation where rampant HGT totally erases the historical signal and makes any attempt to “*accurately reconstruct ancestral genome history*” moot, is possible although the main effect of HGT is shrinking rather than expansion of the ancestral gene set. However, observation of real phyletic patterns makes this possibility highly unlikely. In particular, the prevalence of single-gain patterns implies an uncanny match between the pattern-inferred and sequence-inferred histories which is improbable under the HGT-saturated model.*

Reviewer response: In my application of ml ancestral state reconstruction, gene presence in the ancestral state often is favored, because the gene gain and loss are assumed to be constant throughout the tree. However, this often made assumption is almost certainly false. One possible reason is that genes, which were invented later in evolution, cannot have been gained early on. I did not yet test the program used by the authors; however, I suspect that the use of too simple a model assuming uniformity in rates might be the reason for the observed complex archaeal ancestor. It certainly is possible that evolution went from complex to simple inside the archaeal domain; however, analysis of phyletic patterns alone in the absence of a test for the impact of model misspecification, and without corroborating phylogenetic evidence, only results in a preliminary finding that remains highly questionable.

Authors’ response to reviewer’s response: *Fortunately, the model behind the COUNT program is more sophisticated than that. First, the gain and loss rates are not assumed to be constant, but are estimated for the tree branches separately. Second, the deep ancestral state is always set to zero and the gain on the branch leading to the LCA is no more favored than the data dictates. Taking into account the data beyond the phyletic patterns (individual gene phylogenies) might, in principle, provide even better results; unfortunately, this is currently beyond practical state of the art as we understand it.*

3) In Figure 1, the boundary between which proteins belong to the shell and which belong to the variable cloud is somewhat fuzzy. The authors should comment on the following. Depending upon where one sets this boundary, how would it affect the size estimation of the ancestral archaeal genome (LACA)? Similarly, would lumping together non-identical patterns artificially increase the size of LACA?

Authors’ response: *The boundary between the shell and the cloud, as well as that between the core and the shell, includes uncertainty (is fuzzy) by definition. The reported numbers are the integrals of the “core”, “shell” and “cloud” exponents, not a result of counting families within arbitrarily defined boundaries. Redefining these boundaries would have no effect on LACA size and content estimates because the estimates are derived from specific phyletic patterns regardless of the arbitrary “core”, “shell” and “cloud” status.*

We are not sure about the meaning of the rest of this comment. We do not lump patterns together whether they are identical or non-identical; neither can we see any rationale for doing so. Therefore we cannot venture to guess how this procedure would affect the LACA size estimate.

Reviewer response: In lumping patterns together, I was referring to the sentence in the text where you say “The rapidly increasing proportion of unique phyletic patterns calls for a more coarse-grained comparison whereby non-identical but similar patterns are treated as members of the same group”. My concern is that depending on which cutoff you use to determine if patterns are similar enough to be considered as part of the same group, there is a possibility that those patterns, if analyzed individually, may yield different results, or at least have different probabilities of being present in LACA.

Authors’ response to reviewer’s response: *There was no “lumping” with arbitrarily set cutoffs to “*determine if patterns are similar enough to be considered as part of the same group*”. All patterns were analyzed individually. The required “coarse-graining” of the analysis emerged naturally by concentrating on the more biologically interesting patterns of inferred gains and disregarding the less interesting pattern of losses.*

4) The final claim of a LACA with ~2600 genes is a little optimistic. This number is based on the ML estimated genome size of ~1725 arCOGs, of which only about half have a p-value > 90%, with arbitrarily added numbers of paralogs and transient genes. There should be a better explanation of how these numbers were determined and how confident the authors about the conclusions.

Authors’ response: *In the extant genomes, on average, the ratio between the number of genes in the genome and the number of detected arCOGs is approximately 1.5. Because we do not have any reason to believe that LACA was qualitatively different from modern Archaea, we used this ratio to arrive to ~2600 genes from ~1725 ancestral arCOGs. The latter number does not depend on the confidence level assigned to each particular gene family but rather represent the sum of posterior probabilities. For example, 8 genes each with a 25% chance of being present in LACA, together would contribute ~2 genes to the estimate of the LACA family set.*

Reviewer response: I think one should be cautious in making assumptions about the state of LACA based on observation from modern genomes without corroboration using other methods than phyletic patterns alone. You arrive at a number of about 1725 ancestral arCOGs, of which only about 850 arCOGs can be traced back to the ancestral genome with good confidence level. The other halves are not well substantiated extrapolation. In addition, if you are overestimating the number of arCOGs in LACA because of flaws in the model used, the size of LACA would be even smaller. The best thing to do in my opinion would be to build phylogenetic trees from these arCOGs and to compare them to the reference phylogeny to determine if they follow a vertical line of descent or not. Only then could one make reasonable conclusions about ancestral genome content.

Authors’ response to reviewer’s response: *Not* “making assumptions about the state of LACA” *was exactly our motivation. As mentioned earlier, we have no reason to believe that LACA was qualitatively different from modern Archaea; thus, the natural assumption about LACA is that it is similar. The notion that only 850 genes are “*confidently*” (i.e. with posterior probability exceeding some arbitrary cutoff) traced to LACA is not quite relevant. In fact, 1725 families is the maximum likelihood estimate of LACA size regardless of which (and how many) individual gene families contributed what fraction of posterior probability to the final estimate. Again, detailed analysis of individual gene histories might improve the estimates for individual genes but unless the collective shift in these estimates would turn out to be massively asymmetric, the overall estimate would change very little.*

5) On the phylogenetic tree in Figure 2, it would be advisable to add support values to the tree to have a better idea of the reliability of the reference phylogeny since any misplaced branches can greatly influence the final results.

Authors’ response: *First, fortunately, misplaced branches tend to have only a minor effect on reconstructions because of the tendency of reconstruction errors to be confined to short internal branches where the number evolutionary events is usually small. Rearrangement of such branches affects the inference of events only minimally and locally. Second, given the size of the concatenated ribosomal protein alignment, confidence levels for the tree branches are mostly inflated. In the tree in Figure*2*, all branches have reported bootstrap support values >0.9 which is most likely overly optimistic. The sole exception is the branch of* Methanococcales *whose position relative to its presumed sister group and to* Methanobacteriales *is effectively unresolved (support value of 0.23).*

6) Figure 4. Do the different box sizes on the tips of the tree having any meaning? If so, please explain.

Authors’ response: *Single-size (square) boxes are used for branches ending in single genomes and is always colored uniformly. Double-size (rectangular) boxes are used for compressed clades and usually contain the color transition between the smallest and the largest genome in the clade. We added this information to the figure legend.*

7) Figure 6. Is there any relevance to the different line colors depicting the byways of gene sharing? There are a few byways that seem to defy logic. One example is the byways linking the Methanomicrobiales to their own ancestor near the base of the tree. The authors should explain the cause of a set of genes travelling back or forward in time.

Authors’ response: *The line colors in Figure*6*are not informative and are used for better visual separation only (we added the explanation to the figure legend). It should be noted that byways shown in this picture connect the nodes where gains have occurred; they do not depict the actual transfer paths (the latter cannot be derived from phyletic patterns in principle). A connection between an ancestor and its descendant is inferred when a gene family appears to be gained in some deep clade, lost subsequently, and then re-gained by a shallow descendant of the same clade. Any member of the donor clade, extant or extinct, that is contemporary to the ancestor of the acceptor clade, can be the source of the transfer. Again, the exact path cannot be derived from phyletic patterns only.*

## Supplementary Material

Additional file 1 **The archaeal ribosome phylogeny.** The phylogenetic tree reconstructed for concatenated alignments of ribosomal proteins using FastTree program, Newick format. Click here for file

Additional file 2 **The inferred LACA genome content.** Posterior probabilities for the presence of an arCOG in LACA genome. Click here for file

Additional file 3 **Gains and multiple gains on the archaeal tree branches.** The sums of posterior gain probabilities for all arCOGs and arCOGs involved in multiple gain events for the archaeal tree branches (node numbers correspond to labels in the Additional file 1). Click here for file

Additional file 4 **High-frequency two-gain patterns.** The observed and expected numbers of the two-gain patterns (node numbers correspond to labels in the Additional file 1; p-value computed using expectation under the Poisson distribution). Click here for file
